# Dysregulated lncRNAs in Cisplatin-Induced Nephrotoxicity and Their Association with Apoptosis and Autophagy: An Exploratory In Vitro Study

**DOI:** 10.3390/ijms262211201

**Published:** 2025-11-19

**Authors:** Yuliannis Lugones, Pía Loren, Carola E. Matus, Nelia M. Rodriguez, Pamela Leal-Rojas, Rody San Martín, Kathleen Saavedra, Nicolás Saavedra, Patricia Moriel, Luis A. Salazar

**Affiliations:** 1 Doctoral Program in Sciences Major in Applied Cellular and Molecular Biology, Universidad de La Frontera, Temuco 4811230, Chile; yuliannis1984@gmail.com; 2 Center of Molecular Biology and Pharmacogenetics, Department of Basic Sciences, Faculty of Medicine, Universidad de La Frontera, Temuco 4811230, Chile; ploren@uct.cl (P.L.); carola.matus@ufrontera.cl (C.E.M.);; 3 Laboratorio de Investigación en Salud de Precisión, Departamento de Procesos Diagnósticos y Evaluación, Facultad de Ciencias de la Salud, Universidad Católica de Temuco, Manuel Montt 056, Temuco 4780000, Chile; 4 Departamento de Ciencias Biológicas, Instituto de Ciencias Biomédicas, Facultad de Ciencias de la Salud, Universidad Autónoma de Chile, Temuco 4810101, Chile; nelia.rodriguez@uautonoma.cl; 5 Center of Excellence in Translational Medicine (CEMT) & Scientific and Technological Bioresource Nucleus (BIOREN), Universidad de La Frontera, Temuco 4810296, Chile; pamela.leal@ufrontera.cl; 6 Molecular Pathology Laboratory, Institute of Biochemistry and Microbiology, Science Faculty, Universidad Austral de Chile, Valdivia 5110566, Chile; 7 School of Medical Sciences, University of Campinas, Campinas 13083887, SP, Brazil; patricia.moriel@fcf.unicamp.br; 8 Faculty of Pharmaceutical Sciences, University of Campinas, Campinas 13083871, SP, Brazil

**Keywords:** cisplatin, nephrotoxicity, lncRNA, apoptosis, autophagy

## Abstract

Cisplatin is a widely used chemotherapeutic agent, but its clinical application is limited by nephrotoxicity. Conventional renal markers lack sensitivity for early cisplatin nephrotoxicity while long non-coding RNAs (lncRNAs) display cisplatin-responsive changes with exploratory value. The present study aimed to explore the differential expression of eight lncRNAs on in vitro model of cisplatin-induced nephrotoxicity. Human kidney cell lines HEK-293 and HK-2 were exposed to increasing concentrations of cisplatin for 24 h. Cell viability was determined by colorimetric assays to ascertain the concentrations resulting in 25% (IC_25_), 50% (IC_50_), and 75% (IC_75_) cell death (inhibitory concentration). Apoptotic and autophagy-related proteins were analyzed by Western blot, and reverse transcription–polymerase chain reaction was employed to evaluate the expression of the lncRNAs. Cisplatin-induced cell death with IC_25_, IC_50_, and IC_75_ values of 8.8, 15.43 and 27 μM for HEK-293 cells, and 8.1, 13.57, and 22.8 μM for HK-2 cells. Protein analysis showed an increase in cleaved caspase-9, reduction of caspase-3 and increased LC3-II/LC3-I ratio, with no changes in caspase 7 and Beclin-1. The lncRNAs UCA1, XLOC_032768, HOTAIR, LINC-ROR, and PRNCR1 were downregulated, whereas OIP5-AS1 was upregulated; in contrast, GAS5 and PVT1 remained unchanged. In conclusion, this exploratory in vitro study identifies cisplatin-responsive dysregulation of lncRNAs in human renal cells and delineates their associations with apoptosis and autophagy.

## 1. Introduction

Cancer is the second leading cause of death worldwide, accounting for one in every six deaths [1]. In Chile, during 2022, a total of 31,440 deaths related to different types of neoplasms were reported [2,3]. Cisplatin (cis-diaminodichloroplatinum (II), CDDP) is a successful antineoplastic drug used in the treatment of different types of solid organ cancers, including head and neck, testicular, small and non-small cell lung, ovarian, cervix, bladder, among others [4,5,6,7,8,9,10]. However, the clinical use of cisplatin is severely limited by nephrotoxicity, which develops in at least 30% of patients [11]. Cisplatin-induced nephrotoxicity (CIN) can occur in several forms, but the most frequent and severe being acute kidney injury, with an incidence ranging from 20 to 30% after a single dose [12]. Moreover, repeated cycles predispose patients to lifelong dialysis and a high risk of medium- and long-term mortality [13]. Although preventive strategies such as optimized hydration, dosing algorithms and adjunctive nephroprotective agents have been implemented, CIN continues to be the most relevant dose-limiting toxicity of cisplatin [14]. Furthermore, early detection of cisplatin-induced nephrotoxicity (CIN) remains challenging because conventional renal indicators are insufficiently sensitive at the onset of injury, when clinical decisions could still mitigate harm. Nevertheless, despite extensive reports on the cellular and molecular mechanisms through which cisplatin induces nephrotoxicity, the contribution of lncRNAs to this adverse effect remains largely underexplored.

Long non-coding RNAs (lncRNAs) are protein non-coding RNAs with more than 200 nucleotides that alter chromatin structure and DNA accessibility through histone modification and DNA methylation [15,16]. They play a prominent role in several biological processes through modulation of gene expression, including cell differentiation and immune responses [16,17]. Currently, research strategies for studying lncRNAs focus on their identification, characterization, functional roles, and underlying molecular mechanisms in disease [18].

The lncRNAs UCA1 and PVT1 have been implicated in the control of apoptosis, in part by suppressing caspase-3 expression and thereby attenuating the execution phase of apoptosis [19,20]. Within this apoptotic framework, inhibition of LINC-ROR reduces proliferation and migration while increasing apoptosis, whereas its enrichment promotes chemoresistance in part by dampening p53 signaling [21]. Extending this axis toward autophagy, HOTAIR regulates autophagy via ATG-family targets, thereby linking survival pathways with autophagic control [22]. In the context of tubular injury, XLOC_032768 attenuates cisplatin-induced apoptosis and TNF-α–mediated inflammatory responses [23]. Similarly, PRNCR1 decreases apoptosis through the miR-182-5p/EZH1 axis, and OIP5-AS1 is associated with lower levels of cleaved caspase-3/9 and Bax [24,25]. In contrast, GAS5 promotes apoptosis, at least in part via inhibition of miR-205-5p [26].

Understanding whether specific lncRNA responses to cisplatin occur in human renal epithelial cells could inform future mechanistic studies and, ultimately, preclinical and clinical investigations [27]. Therefore, the present study aims to evaluate the differential expression of eight lncRNAs—UCA1, XLOC_032768, HOTAIR, LINC-ROR, PRNCR1, OIP5-AS1, GAS5, and PVT1—in an in vitro model of CDDP-induced nephrotoxicity.

## 2. Results

### 2.1. Determination of the Half-Maximal Inhibitory Concentration (IC_50_)

**Figure 1:**
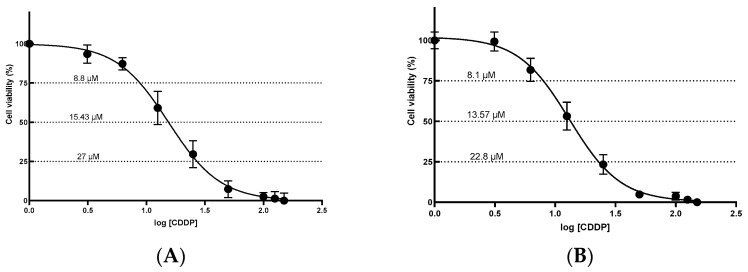
Cytotoxic effect induced by cisplatin (**A**) HEK-293 and (**B**) HK-2 cells. Cells were treated with different concentrations of cisplatin for 24 h. Data are expressed as the mean ± standard error of the mean (SEM) for at least five replicates. CDDP: cisplatin; SEM: standard error of the mean.

To evaluate cisplatin cytotoxicity in renal cells, HEK-293 and HK-2 cell lines were exposed to increasing concentrations of the chemotherapeutic agent (0–150 μM) for 24 h. As shown in Figure 1, cisplatin significantly reduced cell viability in both lines, exhibiting a clear concentration-dependent cytotoxic effect (Figure 1A and Figure 1B, respectively). In both models, the viability curves displayed a characteristic sigmoidal dose–response as drug concentration increased.

The half-maximal inhibitory concentrations (IC_50_) were determined by nonlinear logistic regression using GraphPad Prism v8.0.1. From the fitted dose–response curves, we also interpolated the concentrations required to produce 25% and 75% inhibition of cell viability (IC_25_ and IC_75_, respectively), as reported in Table 1. Model fit was excellent in both cases (R^2^ > 0.980). The estimated IC_50_ values were 15.43 µM for the HEK-293 cell line and 13.57 µM for HK-2 (Table 1).

### 2.2. Characterization of Apoptosis and Autophagy in HEK-293 and HK-2 Cells Exposed to Cisplatin

To delineate the molecular response to cisplatin in a comparative manner, canonical markers of apoptosis (caspases 3, 7, and 9) and autophagy (LC3 and Beclin-1) were analyzed in HEK-293 and HK-2 cell lines after 24 h of exposure to graded drug concentrations.

**Figure 2:**
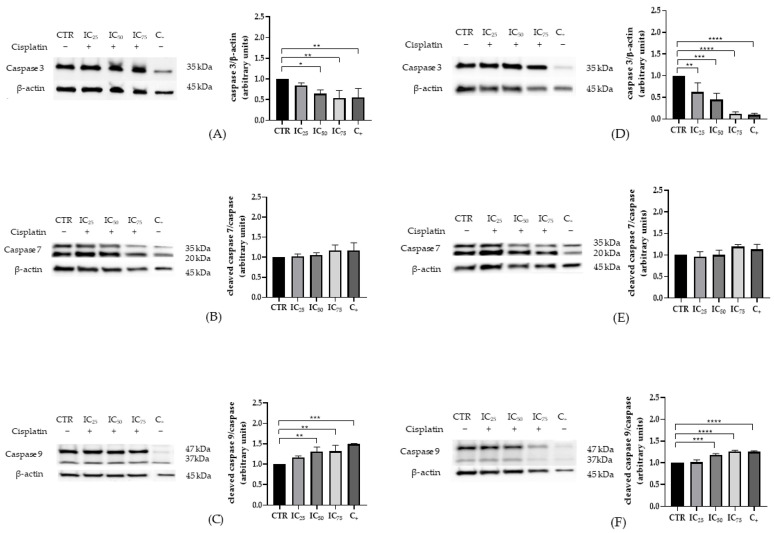
Expression of apoptotic proteins in HEK-293 and HK-2 cells after 24 h exposure to cisplatin at IC_25_, IC_50_, and IC_75_ doses derived from 24 h viability assays (HEK-293: 8.8, 15.43, 27 μM; HK-2: 8.1, 13.57, 22.8 μM, respectively). Representative Western blot and densitometric quantification of (**A**,**D**) Total caspase-3, (**B**,**E**) cleaved caspase-7 and (**C**,**F**) cleaved caspase-9 for HEK-293 and HK-2, respectively, and normalized to β-actin. A 10% DMSO condition was included as a positive control for cell death. Data are presented as mean ± standard error of the mean (SEM) from three independent biological replicates. Statistical analysis: one-way ANOVA (analysis of variance) followed by Tukey’s multiple-comparisons test (* *p* < 0.05, ** *p* < 0.01, *** *p* < 0.001 and **** *p* < 0.0001).

In HEK-293 cells, a clear, dose-dependent activation of the intrinsic pathway was observed, evidenced by a progressive increase in cleaved caspase-9 both by intensification of the bands corresponding to the active fragments (37 kDa) and by higher normalized densitometric signal relative to control (Figure 2C). In parallel, total caspase-3 decreased gradually as the cisplatin dose increased (Figure 2A). By contrast, cleaved caspase-7 remained stable across the entire concentration range, without systematic deviations from control (Figure 2B). Consistently, HK-2 cells reproduced this pattern with a dose-dependent rise in cleaved caspase-9 (Figure 2F) and a concomitant reduction in total caspase-3 (Figure 2D). Notably, the magnitude of these changes was greater and appeared at lower concentrations than in HEK-293 at equivalent doses, indicating higher susceptibility of HK-2 to cisplatin at 24 h. As in HEK-293, cleaved caspase-7 showed no appreciable variation versus control (Figure 2E).

Taken together, both models display a profile consistent with activation of cisplatin-induced intrinsic apoptosis characterized by increased cleaved caspase-9 and decreased total caspase-3 with no evidence of changes in cleaved caspase-7, and with a larger overall response in HK-2 than in HEK-293.

**Figure 3:**
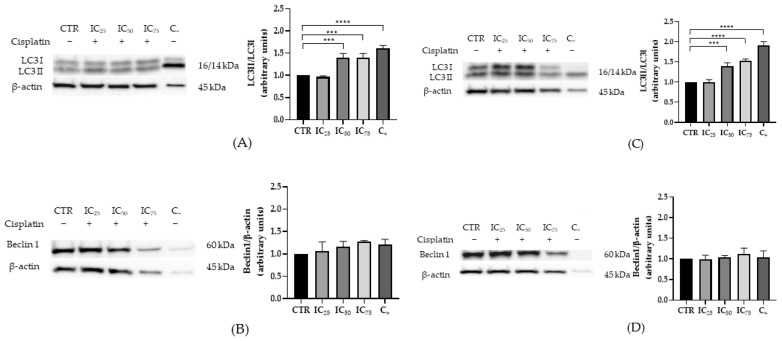
Expression of autophagy-related proteins in HEK-293 and HK-2 cells exposed to increasing concentrations of cisplatin for 24 h and 10% DMSO as a positive control for cell deaths assessed by Western blot. Representative Western blot and densitometric quantification of LC3-I/II (**A**,**C**) and Beclin-1 (**B**,**D**) for HEK-293 and HK-2, respectively, and normalized to β-actin. Data are presented as mean ± standard error of the mean (SEM) from three independent biological replicates. Statistical analysis: one-way ANOVA (analysis of variance) followed by Tukey’s multiple-comparisons test (*** *p* < 0.001 and **** *p* < 0.0001).

Regarding autophagy, both cell lines exhibited a dose-dependent increase in the LC3-II/LC3-I ratio, with clear differences from control at intermediate concentrations and maximal changes at the highest doses (Figure 3A,C). In contrast, total Beclin-1 levels remained unchanged across the exposure range in both HEK-293 and HK-2 (Figure 3B,D).

In summary, these data delineate a scenario in which activation of intrinsic apoptosis reflected by higher cleaved caspase-9 alongside lower total caspase-3 coexists with enhanced LC3 processing, with a steeper sensitivity gradient in HK-2. This dose-dependent pattern at 24 h supports the robustness of the experimental model for comparative studies of cisplatin cytotoxicity and adaptive responses in human renal epithelium.

### 2.3. LncRNA Expression in a Cisplatin-Induced Nephrotoxicity Model

**Figure 4:**
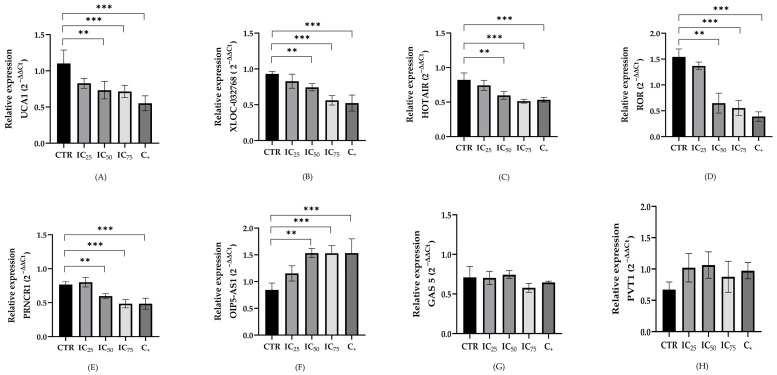
Expression of long non-coding RNAs UCA1 (**A**), XLOC_032768 (**B**), HOTAIR (**C**), LINC-ROR (**D**), PRNCR1 (**E**), OIP5-AS1 (**F**), GAS5 (**G**) and PVT1 (**H**) in HEK-293 cells treated with different concentrations of cisplatin (IC_25_= 8.8 μM, IC_50_= 15.43 μM, and IC_75_ = 27.0 μM) and 10% DMSO as a positive control for cytotoxicity. Expression was normalized using U6 as an endogenous control. Data are expressed as means ± standard error of the mean (SEM) from four independent biological replicates, with each measurement performed in duplicate. Statistical analysis was performed using analysis of variance (ANOVA) and Tukey’s post-test (** *p* < 0.001, and *** *p* < 0.0001).

**Figure 5:**
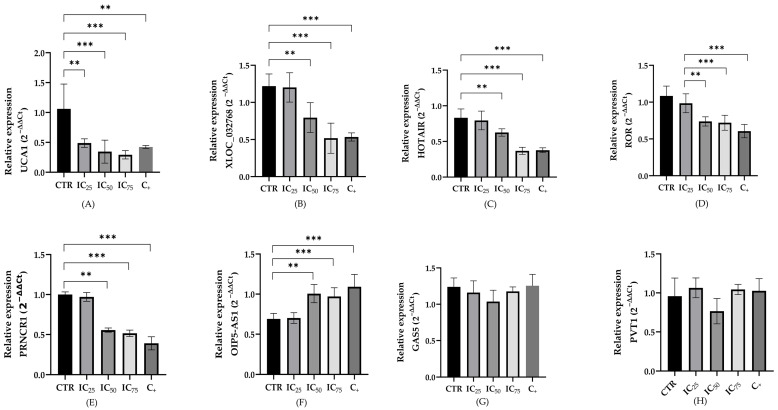
Expression of long non-coding RNAs UCA1 (**A**), XLOC_032768 (**B**), HOTAIR (**C**), LINC-ROR (**D**), PRNCR1 (**E**), OIP5-AS1 (**F**), GAS5 (**G**) and PVT1 (**H**) in HK-2 cells treated with different concentrations of cisplatin (IC_25_ = 8.1 μM, IC_50_ = 13.57 μM, and IC_75_ = 22.8 μM) and 10% DMSO as a positive control for cytotoxicity. Expression was normalized using U6 as an endogenous control. Data are expressed as means ± standard error of the mean (SEM) from four independent biological replicates, with each measurement performed in duplicate. Statistical analysis was performed using analysis of variance (ANOVA) and Tukey’s post-test (** *p* < 0.001 and *** *p* < 0.0001).

The relative expression levels of the lncRNAs were evaluated in both cell lines following cisplatin exposure. Figure 4 and Figure 5 illustrate the expression levels of these eight lncRNAs after 24 h of treatment under the following conditions: untreated cells (control), concentrations of 8.8, 15.43 and 27 µM of cisplatin for HEK-293 and 8.1, 13.57, and 22.8 µM of cisplatin for HK-2 (CI_25_, CI_50_, and CI_75_, respectively), and cells treated with 10% DMSO as a positive control for cell death.

The results revealed significant differences in the expression levels of the five lncRNAs (UCA1, XLOC, HOTAIR, LINC-ROR and PRNCR) after treatment in at least two of the assessed concentrations in both cell lines. These lncRNAs exhibited a significant decrease in their expression compared to the control. In contrast, the lncRNA OIP5 showed an increment in the level expression increase, being statistically significant from the CI_50_ (13.57 µM and 15.43 µM) for HEK-293 and HK-2, respectively. The lncRNAs GAS5 and PVT1 maintained similar expression levels across all evaluated doses.

A comparative overview of the expression changes in the eight lncRNAs under different treatment conditions is shown in Table 2.

## 3. Discussion

Nephrotoxicity is the principal adverse effect of cisplatin therapy, occurring in 20–30% of treated patients and causing rapid deterioration of renal function [28]. This adverse effect may progress to chronic kidney impairment and is associated with increased morbidity and mortality, even when preventive strategies are implemented [11,12].

Human cell-based in vitro toxicity assays are reshaping safety assessment and mechanistic toxicology by integrating high-throughput readouts of cell viability and apoptosis, membrane integrity, nuclear receptor activity, and toxicity pathways [29]. Two widely validated cell lines were used. HK-2 cells, derived from the human proximal tubular epithelium, preserve key morpho-functional attributes of the in vivo proximal tubule and are a reference for drug-induced nephrotoxicity studies [30,31]. HEK-293, in turn, is a versatile human platform in cell biology and toxicological evaluation due to high transfectability, rapid growth, ease of culture, and capacity to execute human post-translational modifications, making it suitable for interrogating signaling pathways and cytotoxic responses [32]. Several recent studies pair HEK-293 in vitro assays with in vivo validation in cisplatin nephrotoxicity models—for example, morin hydrate demonstrated protection in HEK-293 and was subsequently confirmed in mouse kidney while 4-hydroxychalcone showed cytoprotection in HEK-293 accompanied by in vivo genotoxicity/renal readouts [33,34]. These exemplars illustrate the common workflow in which HEK-293 mechanistic signals (apoptosis/oxidative stress/autophagy) are first defined in vitro and then corroborated in vivo.

In recent years, evaluation of renoprotective properties against cisplatin has often relied on HEK-293 cells as an in vitro model. Protocols commonly use 20 μM cisplatin for 24 h; depending on the design, this exposure reduces viability by 40% up to 75%, a dynamic exploited to detect cytoprotective effects and delineate mechanisms of injury [35,36,37,38]. Consistently, reported 24 h IC_50_ values in HEK-293 cluster around 19–20 μM, although higher concentrations (≥50–100 μM) are also used when robust cytotoxicity is required to probe mechanisms [33,34,39]. In our experimental system, the 24 h IC_50_ was 15.43 μM, close to the range typically described for cisplatin nephrotoxicity assays in HEK-293 cells.

In HK-2 cells, exposure ranges vary across studies, but 20 μM for 24 h is widely employed to elicit reproducible cytotoxicity and to test putative renoprotective interventions. Under this condition, multiple agents have been shown to mitigate acute injury, oxidative stress, and apoptosis in cisplatin-exposed HK-2 cells [40,41,42,43,44]. Work focused on lncRNAs has also used 10 μM cisplatin to analyze modulation of apoptosis and inflammation [25,45]. In our experiments, the 24 h IC_50_ in HK-2 cells was 13.57 μM, which lies within the commonly reported range for this model. In line with prior reports, reductions in nephrotoxicity have been observed at mean exposures of 11.63 μM and 16 μM, while some studies employ higher concentrations (e.g., 40 μM) to induce marked cytotoxicity and examine mechanisms with greater resolution [46,47,48].

Our results confirm that cisplatin produces marked cytotoxicity in human renal lines HEK-293 and HK-2, evident as a dose-dependent decrease in viability after 24 h. This behavior accords with extensive literature identifying cisplatin nephrotoxicity as a clinical dose-limiting adverse effect, largely attributable to preferential drug accumulation in the proximal tubular epithelium [49,50]. HK-2 exhibited greater susceptibility than HEK-293, reflected in lower IC_50_ values. This difference is biologically plausible: HK-2 derives from human proximal tubule and retains renal epithelial functions, including expression of the organic cation transporter OCT2, which facilitates cisplatin uptake and thereby its toxicity [51,52]. However, multiple studies have evaluated HK-2 and HEK-293/HEK-293T in parallel under cisplatin exposure, showing qualitatively similar injury patterns despite differences in transporter expression (e.g., OCT2). Dose- and time-dependent loss of viability with apoptotic/oxidative readouts has been reported in both models, and comparative experiments document aligned apoptosis/necrosis metrics with pharmacological modulation after injury (e.g., Cilastatin) in HK-2 and HEK-293T [53,54,55]. Taken together, these observations support the use of both HK-2 and HEK-293/HEK-293T as complementary human renal models for studying cisplatin-induced injury.

### 3.1. Expression of Apoptotic and Autophagic Proteins in HEK-293 and HK-2 Cells Exposed to Cisplatin

In widely used human renal cell models such as HEK-293 and HK-2, cisplatin activates a predominantly mitochondrial apoptotic program that integrates genotoxic injury, bioenergetic dysfunction, and outer-mitochondrial membrane permeabilization, with sequential activation of the caspase-9, caspase-3 axis in tubular epithelium [56,57]. Our data are concordant: both lines showed increased cleaved caspase-9, decreased total caspase-3, and no consistent change in cleaved caspase-7. The rise in cleaved caspase-9 is consistent with cytochrome c release and apoptosome assembly, which drive caspase-9 dimerization/activation and downstream executioner processing [58,59]. Depletion of total caspase-3 serves as an indirect indicator of activation (consumption by proteolytic cleavage to active subunits) when interpreted alongside increased cleaved caspase-9 and corroborated by functional readouts (e.g., DEVDase activity and PARP cleavage) consistently described in cisplatin-treated tubular epithelium [60,61].

Cleaved caspase-7 may remain stable because its functions partially overlap with, but are not identical to, caspase-3; its activation depends on cell type, signal threshold, and stimulus kinetics. In tubular cells, execution typically relies on caspase-3, whereas caspase-7 often plays a subordinate or transient role that may be missed by single-time-point measurements [60,61,62]. Timing is critical: activation of caspases 8, 9, and 3 becomes detectable from approximately 12 h after in vitro cisplatin exposure. p53 modulates both transcriptional responses and downstream amplification of the apoptotic cascade, with cell type dependent contributions from Bax and Bak and from the executioner caspases 6 and 7 [60,61,63,64]. Consequently, transient peaks of caspase 7 may be missed within a 24 h window, without contradicting the predominant executioner profile centered on caspase 3 reported in the literature and observed here [60,61,62,63,64]. Additionally, under certain chemotherapeutic regimens, caspase-3/GSDME-dependent pyroptosis intersects with apoptosis, maintaining caspase-3 as the central execution node without requiring detectable co-activation of caspase-7 [40].

Pathway crosstalk further refines interpretation. The extrinsic death receptor pathway (caspase-8) can converge downstream on caspase-3, reinforcing execution [57,60,63]. In parallel, endoplasmic reticulum stress (UPR) contributes to cisplatin nephrotoxicity via caspase-12 and effectors such as PERK and ATF6, shaping amplitude and kinetics of apoptosis and potentially explaining differences between cell lines or experimental settings [65,66]. These connections do not negate the primacy of the caspase-9/caspase-3 axis; rather, they contextualize why caspase-7 was unchanged under our conditions while caspase-9 and caspase-3 showed the expected directionality [65,66].

Against this background, autophagy emerges as a cytoprotective mechanism. Cisplatin exposure activates early autophagic programs as an adaptive response to mitochondrial and genotoxic stress, delaying or attenuating apoptotic execution [67,68,69]. The observed increase in the LC3-II/I ratio in HEK-293 and HK-2 is consistent with enhanced autophagosome biogenesis and/or increased autophagic flux, phenomena reported across in vitro and in vivo platinum nephrotoxicity models [56,57]. The lack of change in total Beclin-1 does not contradict this interpretation; consensus guidelines emphasize that total Beclin-1 levels need not correlate with global autophagy rate, which often depends on post-translational regulation and steps downstream of nucleation (ATG7/ATG3-LC3 conjugation) better captured by LC3 than by total Beclin-1 [70]. Renal autophagy in cisplatin injury is organized chiefly through AMPK–mTOR–ULK1 and PINK1/Parkin-mediated mitophagy. AMPK activation and mTOR inhibition initiate autophagy (via ULK1) and favor LC3 conjugation, while mitophagy clears damaged mitochondria, lowering oxidative stress—mechanistically explaining increased LC3-II even when Beclin-1 is unchanged [71,72,73]. Moreover, Beclin-1 function depends on its interaction with Bcl-2, so LC3 frequently provides a more sensitive indicator of operational autophagy within specific time windows [74].

### 3.2. LncRNAs Expression in Cisplatin-Treated Renal Cells

Long non-coding RNAs participate in essential physiological processes, and their dysregulation contributes to diverse diseases. Among epigenetic alterations associated with cisplatin nephrotoxicity, the role of lncRNAs remains underexplored. Here, eight lncRNAs (UCA1, XLOC_032768, HOTAIR, LINC-ROR, PRNCR1, OIP5-AS1, GAS5, and PVT1) were examined in cisplatin-induced nephrotoxicity in two renal cell models (HEK-293 and HK-2). UCA1, XLOC_032768, HOTAIR, ROR, and PRNCR1 were significantly downregulated in both lines in response to all three cisplatin doses tested. By contrast, OIP5-AS1 showed consistent overexpression in both models. GAS5 and PVT1 did not change significantly under the conditions studied.

UCA1 (Urothelial Cancer Associated 1), originally described in urothelial carcinoma, has been implicated in renal injury settings beyond cancer. In an in vivo model of cisplatin-induced AKI, UCA1 is upregulated and promotes inflammation by sponging miR-4498 to derepress AKT3; knockdown of UCA1 reduces circulating cytokines and lowers tubular epithelial apoptosis in a T-cell/TEC co-culture system, supporting an immunomodulatory role of the UCA1–miR-4498–AKT3 axis in AKI [75]. Conversely, in diabetic nephropathy and in HK-2 cells under hyperglycemic conditions, UCA1 is downregulated, and its overexpression attenuates apoptosis and inflammasome activation by targeting miR-206; luciferase/RIP assays confirm the UCA1–miR-206 interaction, and functional rescues demonstrate the axis anti-apoptotic effect in tubular epithelium [76]. Our human renal epithelial models (HEK-293 and HK-2), exhibited transcriptional repression of UCA1 after cisplatin exposure, consistent with context-dependent regulation attributable to model system, time-point, and dose/species.

HOTAIR (HOX transcript antisense RNA) is dysregulated across renal injury, and its function is context dependent. In septic/inflammatory models, HOTAIR increases in kidney and in HK-2 cells and promotes tubular apoptosis, whereas knockdown lowers cytokines and injury indices [77]. In fibrotic remodeling, pharmacologic interventions such as paeonol blunt the HOTAIR–miR-124–Notch axis in both in vitro and in vivo models [78]. Hyperglycemia also links HOTAIR to injury; in HK-2 cells, HOTAIR drives apoptosis via the miR-126-5p/AKT pathway [79]. In diabetic kidney disease, HOTAIR is elevated in human and murine glomeruli, yet podocyte-specific deletion yields a minimal phenotype, underscoring cell-type and context dependence [80]. In our cisplatin nephrotoxicity experiments, HOTAIR decreased in a dose-dependent manner, consistent with context-dependent regulation across renal injury modalities, cell lineages, and exposure conditions.

LINC-ROR (Regulator of Reprogramming) has been implicated in cardiorenal conditions. In patients with heart failure combined with acute renal failure, dysregulation of ROR shows a negative correlation with miR-125b and is associated with higher mortality and rehospitalization [81]. LINC-ROR acts as a negative regulator of p53 signaling, reducing its transcriptional activity and influencing cell-cycle arrest (e.g., p21) and apoptosis in several models; LINC-ROR reduction/silencing restores p53 function and enhances programmed cell death, and recent work reinforces its role as a regulatory node in oncogenic survival pathways [82,83,84]. Reviews focused on renal injury emphasize the central role of p53 in cisplatin nephrotoxicity, lending biological plausibility to LINC-ROR–p53 axes in tubular epithelium [85]. In line with this framework, we observed decreased LINC-ROR expression after cisplatin treatment.

For XLOC_032768, available evidence indicates cytoprotection against oxidative stress with attenuation of cisplatin-induced apoptosis and modulation of TNF-α mediated inflammatory responses in tubular epithelium [23,45]. Consistently, we found significant downregulation of XLOC_032768 in cisplatin-treated HEK-293 and HK-2. This accords with reports of XLOC_032768 downregulation in mouse kidneys after ischemia–reperfusion and in hypoxic renal cells; therapeutic XLOC_032768 preserved cell viability and tissue integrity, suggesting a positive contribution to tubular anti-apoptotic capacity and to renal repair [45].

Recent reviews position PRNCR1 (Prostate cancer non-coding RNA within lncRNA–miRNA networks linked to apoptosis and inflammation in AKI [24,86]. In cisplatin-induced AKI models, PRNCR1 expression decreases, while its overexpression reduces renal epithelial apoptosis and improves viability via the miR-182-5p/EZH1 axis [87]. Our results indicate that, PRNCR1 was reduced by cisplatin in HEK-293 and HK-2, mirroring prior reports [24,86,87].

OIP5-AS1 (OIP5 Antisense RNA 1) has been associated with pro-survival responses and chemoresistance. In the kidney, OIP5-AS1 lowers apoptosis in cisplatin AKI via the miR-144-5p/PKM2 axis and is included in AKI-relevant lncRNA–miRNA networks for apoptosis and inflammatory stress [25,87]. Consistent with this profile, in diabetic nephropathy OIP5-AS1 promotes epithelial-to-mesenchymal transition and fibrosis by binding miR-30c-5p, placing this lncRNA as a direct modulator of profibrotic tubular reprogramming [88]. Complementarily, in sepsis-induced kidney injury, the OIP5-AS1/miR-186-5p/NLRP3 axis enhances NLRP3 inflammasome activation, linking OIP5-AS1 to innate inflammation and acute tubular damage [89]. In our model, OIP5-AS1 was overexpressed in cisplatin-exposed HEK-293 and HK-2, consistent with a cytoprotective role in acute renal injury and compatible with an adaptive tubular survival response.

GAS5 (Growth arrest-specific transcript 5) shows context-dependent regulation: in ischemia–reperfusion AKI, GAS5 increases and exerts pro-apoptotic effects by acting as a ceRNA for miR-21 in tubular epithelium, and reviews place GAS5 in lncRNA–miRNA networks integrating apoptosis, oxidative/inflammatory stress, and autophagy in AKI [86,90]. In diabetic nephropathy studies show that GAS5 is reduced in renal tissue and in high-glucose-challenged tubular cells, and that restoring GAS5 attenuates oxidative stress, pyroptosis, and profibrotic signaling through miRNA axes such as GAS5/miR-452-5p and GAS5/miR-221 [91,92]. During fibrotic remodeling, GAS5 constrains TGF-β signaling via the miR-142-5p axis, and loss of Gas5 worsens fibrosis after unilateral ureteral obstruction in mice [93,94]. In sepsis-associated AKI, GAS5 limits tubular pyroptosis by regulating miR-579-3p and activating the SIRT1–PGC-1α–Nrf2 pathway [95]. This apparent heterogeneity may reflect GAS5’s modular architecture, with independent structural domains enabling distinct responses by cell type, microenvironment, dose, and time [96,97]. In this light, our finding of unchanged GAS5 across cisplatin concentrations in HEK-293 and HK-2 is consistent with context-dependent regulation.

PVT1 (Plasmacytoma Variant Translocation 1) is consistently implicated in renal clinical and experimental settings. In diabetic nephropathy, its silencing attenuates podocyte injury and apoptosis through a FOXA1-dependent axis [98]. Genetic evidence supports this link: PVT1 was identified as a candidate gene for end-stage renal disease in type 2 diabetes and PVT1 variants (e.g., rs3931283) associated with diabetic kidney disease and renal function markers in patients with type 2 diabetes [99,100]. In cardiorenal syndrome, PVT1 dysregulation relates to chronic kidney disease progression in patients with congestive heart failure, and functional data suggest that its modulation contributes to worsening renal injury [101]. In human renal epithelium—mainly in non-tumoral, LPS-induced AKI—PVT1 modulates oxidative/inflammatory stress and cell death in HK-2 via the miR-27a-3p/OXSR1 axis, supporting its responsiveness to tubular injury [102]. In our cisplatin model, PVT1 did not change significantly in HEK-293 or HK-2. This pattern is consistent with regulation shaped by cell type, microenvironment, and injury kinetics. Under our conditions, PVT1 does not appear to be a primary mediator of the drug response.

**Figure 6:**
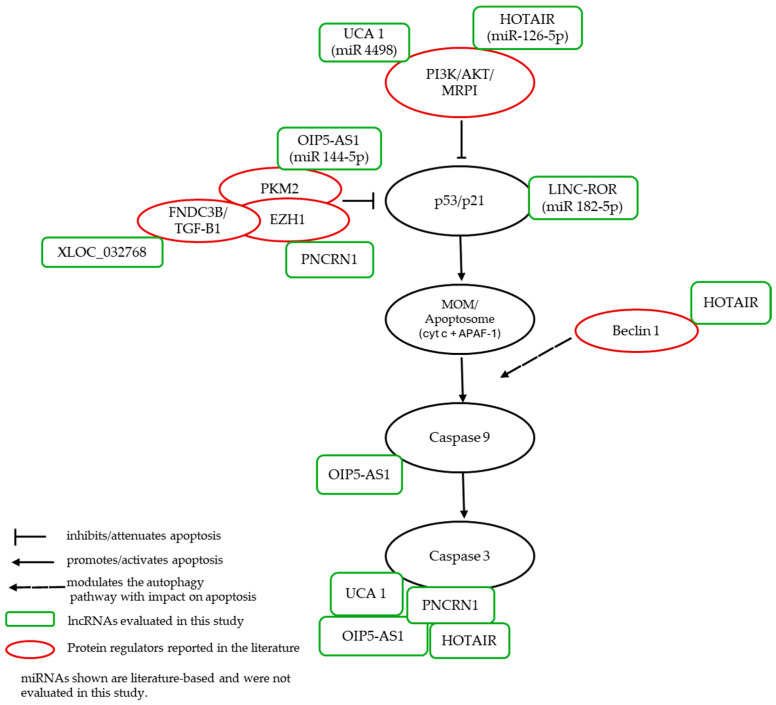
Working model of the mitochondrial apoptosis pathway and its lncRNA modulators in cisplatin-exposed human renal epithelium (in vitro). The scheme summarizes p53/p21 control and the mitochondrial axis (BAX/BAK → MOMP → cytochrome c/APAF-1 → caspase-9 → caspase-3) (black ovals) with protein regulators reported in the literature: PI3K/AKT, EZH1, PKM2, Beclin-1, FNDC3B/TGF-β1 (red ovals). lncRNAs evaluated in this study (green rectangles): UCA1, HOTAIR, LINC-ROR, OIP5-AS1, PRNCR1, and XLOC_032768 are positioned according to literature-supported interactions and miRNAs shown are literature-based and were not evaluated in this study. Key: continuous arrow, promotes/activates apoptosis; continuous arrow with bar (⊣), inhibits/attenuates apoptosis; non-continuous arrow, modulates autophagy with impact on apoptosis.

Finally, to integrate our observations with prior knowledge, we present a literature-based working model that situates the studied lncRNAs onto canonical nodes of apoptosis and autophagy (Figure 6).

Taken together, our in vitro renal epithelial data (HEK-293 and HK-2) delineate a compact cisplatin nephrotoxicity signature integrating mitochondrial apoptosis (cleaved caspase-9 and caspase-3) and adaptive autophagy (elevated LC3-II/I). Our results show cisplatin-responsive expression—especially downregulation of UCA1 and XLOC_032768, supported by HOTAIR, PRNCR1, and LINC-ROR decreases and OIP5-AS1 upregulation—collectively reflecting the apoptosis–autophagy balance characteristic of cisplatin nephrotoxicity. Despite the biological differences between HK-2 and HEK-293, both models showed similar behavior across all experiments performed. This concordance reinforces the cross-model robustness of the responses to cisplatin.

However, this work has limitations. First, the transcriptomic panel was restricted to eight lncRNAs, so additional lncRNAs relevant to cisplatin nephrotoxicity cannot be excluded. Second, in vitro models (HEK-293 and HK-2) limit extrapolation to human renal tissue in vivo, where microenvironment, perfusion, and intercellular interactions (immune and endothelial) modulate drug responses. Third, the use of DMSO as a pro-apoptotic condition may introduce nonspecific effects and should be refined in future designs. Despite these limitations, the findings present a coherent pattern of apoptotic/autophagic activation and lncRNA regulation in cisplatin-exposed renal epithelium and targeted genetic perturbations, and in vivo confirmation to strengthen robustness and translational potential.

## 4. Materials and Methods

### 4.1. Cell Line and In Vitro Culture

The human kidney cell line HEK-293 (Human Embryonic Kidney 293, ATCC, # CRL-1573) and HK-2 (Human Kidney 2, ATCC, #CRL-2190), were cultured in Minimum Essential Medium (MEM, Gibco, Thermo Fisher Scientific, Waltham, MA, USA) and Dulbecco’s Modified Eagle Medium (DMEM-F12, Gibco, Thermo Fisher Scientific, Waltham, MA, USA) supplemented with 10% FBS (Gibco, Thermo Fisher Scientific, Waltham, MA, USA) in humidified atmosphere at 37 °C and 5% CO_2_. HK-2 cells originate from the normal kidney tissue of a healthy adult male. This cell line is widely recognized for its relevance to human renal epithelial biology and is often used to model kidney function and injury in vitro. HEK-293 is a cell line characterized by its epithelial morphology. It was originally isolated from the kidney of a human embryo and is frequently utilized in toxicology research due to its robust growth characteristics and responsiveness to experimental treatments. Assays were performed at 70% confluence of cell growth.

### 4.2. Renoprotection Against Nephrotoxicity

HEK-293 and HK-2 cells were seeded in 24-well plates at a density of 0.05 × 10^6^ cells/mL for 24 h to allow adherence. Subsequently, cells were divided into control groups (no cisplatin, CDDP) and treatment groups exposed to different concentrations of CDDP (P4394, Sigma-Aldrich, Merck Millipore, St. Louis, MO, USA). Dose–response curves for IC_50_ determination were obtained from the MTS assay and analyzed by nonlinear sigmoidal regression using GraphPad Prism software version 8.0.1. Additionally, the inhibitory concentrations for IC_25_ and IC_75_ were calculated through the logistic fit of the sigmoidal curve equation.

$$Y=\frac{100}{\left[1+10ˆ\left[\left(LOG\;IC50-X\right)*HILLSLOPLE\right)\right]}$$

*Y* denotes the expected inhibition (IC_25_ or IC_75_), *X* the cisplatin concentration, and HILLSLOPE the logarithmic slope coefficient. The evaluation of IC_25_ and IC_75_ allowed the characterization of cellular responses at both early and advanced stages of inhibition, providing a more comprehensive overview of cisplatin sensitivity.

Each experiment was performed in technical and biological triplicates and 10% DMSO solution was used as a positive control for cytotoxicity.

### 4.3. Western Blot

Total protein extraction was performed using RIPA solution and protein concentration was determined using the BCA assay. Equal amounts of protein (50 μg per sample) were resolved on 12% SDS-PAGE and transferred to PVDF membranes. Membranes were blocked and incubated overnight with primary antibodies against caspase 3 (1:1000), caspase 7 (1:1000), caspase 9 (1:1000), LC3I-II (1:1000), Beclin 1 (1:1000) and β-actin antibodies (1:2000) (Cell Signaling Technology, Danvers, MA, USA). After washing, membranes were incubated with HRP-conjugated anti-rabbit secondary antibody. Protein bands were visualized and quantified using ImageJ software version 1.48 (NIH, Bethesda, MD, USA). Band intensities were normalized to β-actin. Results are expressed as the mean ± standard deviation (SD) from at least three independent experiments.

### 4.4. Total RNA Extraction and Quantitative Real-Time PCR

Total RNA from each experimental group (n = 6 replicates per group) was extracted using TRIzol™ Reagent (Invitrogen, Thermo Fisher Scientific, Waltham, MA, USA) according to the manufacturer’s instructions. RNA concentration and purity were assessed by spectrophotometry (A_260_/A_280_ ratio) using an Infinite^®^ M200 PRO NanoQuant Tecan (Tecan Group Ltd., Männedorf, Switzerland). cDNA was synthesized from 1 ng of total RNA using the High-Capacity RNA-to-cDNA kit (Applied Biosystems, Foster City, CA, USA). Quantitative PCR of lncRNAs was carried out using Fast SYBR^®^ Green Master Mix (Applied Biosystems, Foster City, CA, USA) on a real-time PCR system. Primer sequences are provided in Table 3. The qPCR was performed in triplicate including no-template controls. Relative gene expression was calculated using the 2^−ΔΔCt^ method with U6 as the endogenous control.

### 4.5. Statistical Analysis

Data were analyzed using GraphPad Prism v8.0.1 (GraphPad Software Inc., San Diego, CA, USA). Normality was tested prior to analysis. Comparisons between multiple groups were performed using one-way ANOVA followed by Tukey’s post hoc test. A *p*-value < 0.05 was considered statistically significant.

## 5. Conclusions

In human renal cell models (HEK-293 and HK-2), cisplatin exposure elicited indicators of intrinsic apoptosis (increased cleaved caspase-9; reduced total caspase-3) and adaptive autophagy (higher LC3-II/LC3-I ratio). The lncRNA profile revealed a differential regulation characterized by the decrease in UCA1, XLOC_032768, HOTAIR, ROR, and PRNCR1, the increase in OIP5-AS1, and the stability of GAS5 and PVT1. These in vitro findings describe candidate lncRNA responses associated with cisplatin-induced injury. Additional research including loss- and gain-of-function perturbations of priority lncRNAs, and assessment in in vivo and clinical settings is required to determine robustness and translational relevance.

## Figures and Tables

**Table 1:** Estimated inhibitory concentrations of cisplatin (IC_25_, IC_50_, and IC_75_) in HEK-293 and HK-2 cell lines.

Cell Line	IC_25_	IC_50_	IC_75_	R^2^
HEK-293	8.8 ± 1.08	15.43 ± 1.08	27.0 ± 1.03	0.9804
HK-2	8.1 ± 0.86	13.57 ± 0.69	22.8 ± 0.70	0.9876

Values are expressed as mean ± standard deviation. The coefficient of determination (R^2^) indicates the goodness of fit of the model to the experimental data.

**Table 2:** Relative expression of long non-coding RNAs (lncRNAs) in HEK-293 and HK-2 cells treated with cisplatin and 10% DMSO as a positive control for cell deaths.

lncRNA	IC_25_	IC_50_	IC_75_	DMSO 10%
UCA1	-	↓	↓	↓
XLOC_032768	-	↓	↓	↓
HOTAIR	-	↓	↓	↓
LINC-ROR	-	↓	↓	↓
PRNCR1	-	↓	↓	↓
OIP5-AS1	-	↑	↑	↑
GAS5	-	-	-	-
PVT1	-	-	-	-

The results reflect the direction of the relative change compared to the basal condition of each cell line. Symbol legend: ↓ decreased expression; ↑ increased expression; - no appreciable change.

**Table 3:** Sequences of primers used to quantify gene expression by RT-qPCR.

LncRNA	Forward Primer (5′-3′)	Reverse Primer (5′-3′)	Reference
LINC-ROR	CTCCAGCTATGCAGACCACTC	GTGACGCCTGACCTGTTGAC	[103]
PVT1	ATAGATCCTGCCCTGTTTGC	CATTTCCTGCTGCCGTTTTC	[104]
UCA1	CTCTCCATTGGGTTCACCATTC	GCGGCAGGTCTTAAGAGATGAG	[19]
HOTAIR	GCAGTGGAATGGAACGGATT	CGTGGCATTTCTGGTCTTGTA	[105]
PRNCR1	CCAGATTCCAAGGGCTGATA	GATGTTTGGAGGCATCTGGT	[24]
GAS5	CTTCTGGGCTCAAGTGATCCT	TTGTGCCATGAGACTCCATCAG	[106]
OIP5-AS1	TGCGAAGATGGCGGAGTAAG	TAGTTCCTCTCCTCTGGCCG	[107]
XLOC_032768	CATTGCCGACAGCACAACATAC	GCCTATTTAGCAGCCCACCTC	[45]
U6	CTCGCTTCGGCAGCACATATAC	GGAACGCTTCACGAATTTGC	[108]

LINC-ROR, Long Intergenic Non-Protein Coding RNA, Regulator Of Reprogramming; PVT1, Plasmacytoma Variant Translocation 1; UCA1, Urothelial Cancer Associated 1; HOTAIR; HOX transcript antisense RNA; PRNCR1, Prostate cancer non-coding RNA 1; GAS5, Growth arrest-specific transcript 5; OIP5-AS1, OIP5 Antisense RNA 1; U6, U6 small nucleus RNA.

## Data Availability

The original contributions presented in this study are included in the article. Further inquiries can be directed to the corresponding author.

## Abbreviations

The following abbreviations are used in this manuscript:

HEK-293	Human Embryonic Kidney 293
HK-2	Human Kidney 2
CDDP	cisplatin
CIN	Cisplatin-induced nephrotoxicity
lncRNAs	long non-coding RNAs
FBS	fetal bovine serum
DMSO	dimethyl sulfoxide
IC_50_	half-maximal inhibitory concentrations
IC_25_ IC_75_	concentrations required to produce 25% inhibition of cell viability concentrations required to produce 75% inhibition of cell viability
RNA	Ribonucleic Acid
cDNA	Complementary Deoxyribonucleic Acid
qPCR	Quantitative Polymerase Chain Reaction
ceRNA	competing endogenous RNA
PARP	Poly (ADP-ribose) Polymerase
mTOR	Mechanistic Target of Rapamycin
ULK1	Unc-51 Like Autophagy Activating Kinase 1
OCT2	Organic Cation Transporter 2
